# Dairy goat demography and Q fever infection dynamics

**DOI:** 10.1186/1297-9716-44-28

**Published:** 2013-04-26

**Authors:** Lenny Hogerwerf, Aurélie Courcoul, Don Klinkenberg, François Beaudeau, Elisabeta Vergu, Mirjam Nielen

**Affiliations:** 1 Department of Farm Animal Health, Faculty of Veterinary Medicine, Utrecht University, Yalelaan 7, Utrecht, 3584 CL, The Netherlands; 2 INRA, UMR1300 Biologie, Epidémiologie et Analyse de Risque en santé animale, BP 40706, Nantes, F-44307, France; 3 Ecole nationale vétérinaire, agroalimentaire et de l’alimentation Nantes-Atlantique, UMR BioEpAR, LUNAM Université, Oniris, Nantes, F-44307, France; 4 Present address: Anses, Laboratoire de Santé Animale, Unité EPI, 23, avenue du Général de Gaulle, Maisons-Alfort, 94706, France; 5 INRA, UR341 Mathématiques et Informatique Appliquées, Domaine de Vilvert, Jouy-en-Josas, 78350, France

## Abstract

Between 2007 and 2009, the largest human Q fever epidemic ever described occurred in the Netherlands. The source was traced back to dairy goat farms, where abortion storms had been observed since 2005. Since one putative cause of these abortion storms is the intensive husbandry systems in which the goats are kept, the objective of this study was to assess whether these could be explained by herd size, reproductive pattern and other demographic aspects of Dutch dairy goat herds alone. We adapted an existing, fully parameterized simulation model for Q fever transmission in French dairy cattle herds to represent the demographics typical for Dutch dairy goat herds. The original model represents the infection dynamics in a herd of 50 dairy cows after introduction of a single infected animal; the adapted model has 770 dairy goats. For a full comparison, herds of 770 cows and 50 goats were also modeled. The effects of herd size and goat versus cattle demographics on the probability of and time to extinction of the infection, environmental bacterial load and abortion rate were studied by simulation. The abortion storms could not be fully explained by demographics alone. Adequate data were lacking at the moment to attribute the difference to characteristics of the pathogen, host, within-herd environment, or a combination thereof. The probability of extinction was higher in goat herds than in cattle herds of the same size. The environmental contamination was highest within cattle herds, which may be taken into account when enlarging cattle farming systems.

## Introduction

Q fever, a zoonotic disease caused by the bacterium *Coxiella burnetii*, has been detected in many host species, with ruminants considered to be the main reservoir [1,2]. In most cases the infection in ruminants is asymptomatic, but occasionally abortions occur, which leads to the shedding of large quantities of bacteria in placentas and birth fluids [3]. Transmission between animals and from animals to humans is believed to occur mainly from the environment, through inhalation of contaminated aerosols [1,2]. Most human clinical infections occur in people handling animals or their products and this was generally not perceived as a major public health problem until 2007, when the Netherlands experienced the largest human Q fever epidemic ever described [4,5]. The number of reported human cases was 168 in 2007, 1000 in 2008, 2354 in 2009, 504 in 2010 and 81 in 2011 [6]. The source of the human epidemic was traced back to dairy goat herds where, within subgroups of several herds during 2005–2009, waves of abortions with an incidence of up to 80% had occurred [5,7-9]. Further details on the abortion patterns in these herds are sparse. Intervention measures included vaccination of dairy goats, followed by culling of all pregnant animals from infected dairy goat herds early 2010 to prevent further shedding into the environment during the following kidding season and thus minimizing human exposure [5,10]. In 2010 and the following years, the number of human cases sharply decreased, suggesting that the intervention measures were effective.

In order to prevent and control future problems with Q fever on goat farms and in humans living close to goat farms, an understanding of the mechanisms driving the abortion storms in goat herds is crucial. Much research has been conducted on Q fever dynamics on cattle farms [11-15], but the Q fever dynamics on goat farms are still poorly understood. In broad terms, while there is some evidence to indicate pathophysiological differences between goats and cattle, the pathogenesis in individual cattle and goats is similar (i.e. reproductive disorders are the main clinical sign, the infection is usually asymptomatic, bacterial shedding occurs mainly through birth products and also through milk, faeces, and other various routes) [16]. Thus while there may be differences in the relative proportion of each shedding route in the whole bacterial shedding, the shedding routes are qualitatively similar. Further, while the rate of abortion may be higher in goats than in cattle, in both species reproductive disorders are the main clinical sign [16]. Therefore, it could well be that the known difference between cattle and goat *C. burnetii* dynamics at the herd level, such as the occurrence of abortion storms solely in goat herds, mainly lies in the population dynamics and farm structure, and not so much the manifestation of the infection in individual cows versus goats. In that case, novel options for Q fever prevention and control might target the host population demographics.

If differences in Q fever abortion patterns for goat and cattle herds are indeed mainly driven by host demographics, the occurrence of abortion waves with large numbers of goats aborting due to Q-fever could result from the fact that goat farms are much larger than cattle farms, and that kidding in goat farms is concentrated in the spring rather than continuous throughout the year. The fact that the abortion waves are such a recent phenomenon in the Netherlands could then be explained by the recent (since the 1990s) major intensification of Dutch goat farming. In 1995, 76 000 goats were housed in the Netherlands, of which 56% stayed in professional dairy goat farms. In 2009, 375 000 goats were housed in the Netherlands, of which 80% stayed on ~350 professional farms with 200–10 000 adult goats each [17,18]. These farms are mainly located in the densely populated province of Noord-Brabant, the province where most human cases were reported. Hence, one of the putative causes of the emergence of Q fever in goats in the Netherlands is the intensive husbandry system in which the goats are kept. However, this has never been investigated.

In this paper we address the question whether the abortion waves in the Dutch dairy goat herds and the difference with Q-fever abortion patterns in cattle herds can be explained by herd demographics alone, rather than by a species specific manifestation of the infection. To this end, we adapted an existing and fully parameterized simulation model for within-herd *C. burnetii* transmission in dairy cattle herds in western France. While keeping disease-related parameters fixed, we changed only the farm size, the yearly kidding pattern, and other demographic aspects to match Dutch goat farms to determine whether abortion waves as observed in the Netherlands could be reproduced. In addition, a sensitivity analysis of the model outcomes was performed to identify additional factors that may also in part explain the observed abortion patterns.

## Materials and methods

A model for the infection dynamics of *C. burnetii* in a French cattle herd developed by A. Courcoul et al. [15] was adjusted to represent the population size, seasonal kidding and other characteristics related to the population dynamics of typical Dutch dairy goat herds and to simulate bacterial spread within the herd.

### The original model for cattle herds in western France

The original model, representing the dynamics of *C. burnetii* transmission in French dairy cattle herds, is a stochastic, individual based model with one-week time steps. This model consists of two parts: (i) an epidemiological model, with a compartmental SIR-like structure, coupled with (ii) a population dynamics model, representing introductions of new animals, culling, lactation, and gestation. Both parts of the model are based on data from western France, on endemically infected dairy cattle herds without clinical signs.

**Figure 1:**
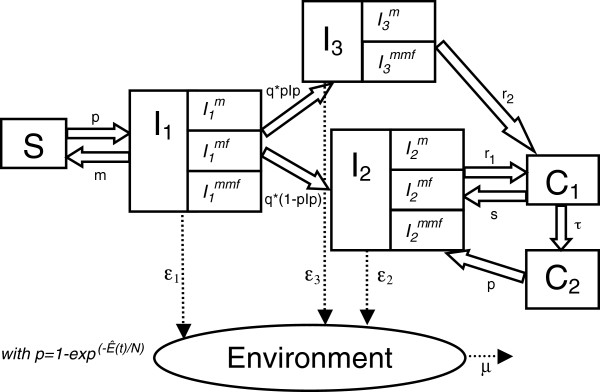
**Flow diagram describing the modeled within-herd spread of *C. burnetii.*** Flow diagram adapted from Courcoul et al. [15], describing the modeled within-herd spread of *C. burnetii*. The health states are the following: *S*, nonshedder apparently susceptible animal, *I*_*1*_, shedder which still has the possibility to eliminate the bacterium and to become *S* again, *I*_*2*_, shedder which no longer has the possibility to become *S* again, *I*_*3*_, shedder which no longer has the possibility to become *S* again and sheds in milk in a persistent way, *C*_*1*_, non-shedder but still infected individual and *C*_*2*_, non-shedder which was *C*_*1*_ in the past but eliminated the bacterium. *I* animals are in the subcategory *m* if they shed in milk only, *mf* if they shed in vaginal mucus/faeces only and *mmf* if they shed in milk and vaginal mucus/faeces. *Environment* represents the environmental bacterial load expressed in dimensionless units, and *p*, the probability of infection or reinfection, is equal to 1 – exp(−*Ê*(*t*)/*N*). *ϵ*_*1*_, *ϵ*_*2*_, and *ϵ*_*3*_ are the quantities of bacteria shed during a time step by an individual *I*_*1*_, *I*_*2*_, and *I*_*3*_ respectively and contaminating the environment. For any shedder, *ϵ* represents the sum, for each shedding route, of the quantity of bacteria released, *Qty*, times *ρ*, its fraction reaching the herd environment. The definitions, values and sources of all parameters in the epidemiological model can be found in Additional file 1.

The epidemiological part of the model is based on a standard SIR-like model (susceptible, infectious, recovered), with added complexity to represent several aspects of *C. burnetii* transmission and shedding patterns that were observed in the field. For a full description of the model see Courcoul et al. [15]. A flow diagram describing the modeled within-herd spread of *C. burnetii* is provided in Figure 1. In brief, susceptible animals (in state *S*) can become infected from the environment (*E*) and move to *I*_*1*_. From *I*_*1*_, the animals can eliminate the bacterium (go back to *S*) or become chronically infected, either without (*I*_*2*_) or with (*I*_*3*_) persistent shedding in milk. In infection states *I*_*1*_, *I*_*2*_, and *I*_*3*_, shedding occurs through different routes (milk and/or mucus/faeces) in different quantities (low, mid, high), which can change from week to week. From *I*_*2*_ and *I*_*3*_, an intermittent shedding pattern can begin by moving to *C*_*1*_ (non-shedding but still infected individuals) and then between *I*_*2*_ and *C*_*1*_. From *C*_*1*_ the animals can eliminate the bacterium and move to *C*_*2*_. From *C*_*2*_, reinfection can take place upon which animals go to *I*_*2*_. During three weeks following infection, reinfection or resumption of shedding of a pregnant animal (i.e. *S*–*I*_*1*_*, C*_*1*_–*I*_*2*_ and *C*_*2*_–*I*_*2*_ transitions), abortion may occur, resulting in temporarily high shedding. Each animal can abort only once in her life. Of all bacteria shed, which are expressed in dimensionless units, a proportion that is dependent on the shedding route enters the environment and thereby contributes to the environmental contamination level *E.* The decay rate of *C. burnetii* in the environment, *μ*, includes the natural mortality of the bacterium and its removal in relation to the periodic cleaning of the barn. All parameters of the epidemiological model with their definitions, values and sources are provided in Additional file 1.

In the population dynamics model, information is represented on the age of the animals, stage of gestation, lactation number and stage of lactation, moment of parturition, moment of introduction into and removal from the herd, for each animal in the herd and for each time step. A description of the model parameters for the herd demography and their values is provided in Table 1.

**Table 1:** Adaptations made to the original cow model to obtain the goat model

**Characteristic**		**Cow model**	**Goat model**
Herd size		50	770
Birth-birth interval		55 weeks	52 weeks
Birth season		year-round	12 weeks
Gestation period		40 weeks	21 weeks
Non-gestation period		15 weeks	31 weeks
Dry period		8 weeks	-
Young animals		no heifers in the model	includes youngstock
Environment		2 environments	1 environment
Replacement rate (year^-1^)		0.355	0.305
Culling rate (week^-1^)	Lactation or age 1	0.0057	0.0029
	Lactation or age 2	0.0052	0.0029
	Lactation or age 3	0.0065	0.0029
	Lactation or age 4	0.0067	0.0136
	Lactation or age 5&6	0.0161	0.0136
	Lactation or age 7&8	NA	0.0136
Probability distribution of the lactation numbers (for cows) or age (for goats) at the start of simulation	Lactation or age 1	0.337	0.285
	Lactation or age 2	0.252	0.245
	Lactation or age 3	0.173	0.211
	Lactation or age 4	0.11	0.136
	Lactation or age 5	0.088	0.067
	Lactation or age 6	0.04	0.033
	Lactation or age 7	NA	0.016
	Lactation or age 8	NA	0.008
Infection from the environment		*p* = 1 – exp(−E(t))	*p* = 1 – exp(−*Ê*(t)/N)
Quantity of bacteria released by shedders in low, mid and high levels respectively, expressed in dimensionless units.		1, 1/30, or 1/3000	1*50, (1/30)*50, or (1/3000)*50
Probability of abortion		0.02	0.02*(40/21)

Description of the model parameters that were adapted in the original cattle model to obtain the default goat model. Parameter definitions and values for the epidemiological model can be found in Additional file 1.

### Adaptation of population dynamics to goat farms in The Netherlands

Goat population dynamics were modeled based on a mean herd, derived from a questionnaire survey, in 2007 in The Netherlands, of 163 goat herds [19]. In the model, it is assumed that every year, 217 new goats are added to the population at uniform random times during a 12-week kidding season. After birth, goats can leave the herd by age-dependent culling rates (Table 1). The yearly new additions of goats and culling rates result in a goat farm of 770 animals on average, with a realistic age distribution and a yearly 30% replacement rate. Pregnancies and resulting deliveries or abortions (related to *C. burnetii* shedding) are modeled independently of the new additions, through “deliveries” that take place in the same 12-week kidding season. This is done for practical reasons, to keep the herd size constant and not to have to simulate birth of males and females and management decision on which females to sell. A “worst case scenario” was chosen for pregnancies and lactation schemes by letting all goats get pregnant every year (maximum potential for bacterial shedding around abortion or parturition) and stay in lactation for the rest of their lives after they have their first parturition, regardless of their gestation stage (maximum potential for shedding in milk). The goats get pregnant at uniform random times during a 12-week mating season, and have a gestation period of 21 weeks. Dutch dairy goats from intensive husbandry systems are kept inside the barn year round (non-grazing system), whilst dairy cattle in western France go outside part of the year (pasture based system). For the model, this has two implications. First, the pasture is not included in the goat model, which means the model has only one environment instead of two (as represented in the cattle model). Second, youngstock are included in the goat model. Young animals are usually kept under the same roof as the lactating goats. Further, since *C. burnetii* has been detected inside the uterus of nulliparous goats from infected Dutch herds [10], this age group is likely to play a role in within-herd transmission dynamics of *C. burnetii*. Because dairy youngstock are usually kept separately from the lactating animals in dairy cattle herds in western France, they were not included in the original model.

### Scaling of transmission rate and abortion rate

A first scaling adjustment had to be made to adjust for the effect of herd size on the probability of infection from the environment (*p*). In the original model, the per capita infection probability in week t, *p*(*t*) is directly related to the amount of bacteria present in the environment by *p* = 1 – *ex*p(−*E*(*t*)), where *E*(*t*) reflects the quantity of bacteria in the environment at time *t*, expressed in dimensionless units [15]. This so called “one hit” Poisson dose response relation assumes every unit of the environment makes an independent contribution to the force of infection, which matches the existing evidence that the infectious dose of *C. burnetii* is likely one bacterium and that animals are exposed via inhalation [20]. Increasing the herd size would result in larger environmental contamination, with *p* increasing up to the point where it reaches the maximum value 1, at which all susceptible animals are infected at each time step. However, the likelihood of infection is not only related to the total amount of bacteria shed into the environment by the animals in the herd. It is also likely to depend on the area that the animals share over which the bacteria are distributed (a larger herd will have a larger area, but not necessarily proportional to the number of animals), and on how easily the bacteria that are present in the environment become available to the animals (possibly depending on the texture of the manure and other factors). There are no data to suggest that for a given prevalence of shedders, a goat in a large herd of goats has a different probability of infection than a cow in a herd of 50 cows. Therefore we rescaled the original per capita environmental contamination level *E*(*t*), and defined a new variable *Ê*(*t*) as the *total* environmental contamination: *Ê*(*t*) = 50 × *E*(*t*), where 50 represents the mean number of animals in the original (cow) herd over the simulation period. Thus, the probability of infection *p* becomes 1 – exp(−*Ê*(*t*)/*N*), where *N* represents the mean number of animals in the modeled cattle or goat herd. Further, the rescaling of the environmental contamination implied that the shedding levels had to be changed accordingly, so they were multiplied by 50 as well.

A second scaling adjustment was needed to accommodate the shorter gestation period of goats. In the model, the abortion rate is a weekly rate, which was calibrated to match field data and expert opinion on abortion rates in dairy cattle in western France, resulting in a certain probability of abortion per pregnancy. In order to have a similar abortion probability per pregnant goat, with a gestation period of 21 instead of 40 weeks, the abortion rate in goats was multiplied by 40/21. An overview of the model adaptations is provided in Table 1.

### Model simulations

Four versions of the model were each run 100 times (100 iterations or herds) over a simulation period of 10 years, as in the original model. In the default version, herd size was 770 goats. The second version used a herd of 50 goats and a yearly addition of 14 individuals to investigate the effect of herd size on within-herd transmission dynamics of *C. burnetii.* The third version was run for 50 cows (the original model), and the fourth for 770 cows, to investigate the effect of seasonal kidding and other goat characteristics.

In addition to these four versions of the model, a sensitivity analysis was performed on the default goat model. For this sensitivity analysis, three elements of the infection dynamics were altered in five scenarios, and one element of the population dynamics was altered in a sixth scenario. The details of these six scenarios are provided below. An overview of the four versions of the model and the six scenarios for the sensitivity analysis of the default model is given in Table 2.

**Table 2:** Description of model variations and sensitivity analyses

**Model**	**Model code**	**Species**	**Herd size**	**Young animals**	**Time introduction infected animal**	**Time kidding introduced infected animal**	**Prob. to abort**	**Prob. of infection**
Variations of the model								
m1	770 goats	Goats	770	Yes	1	1	0.038	1–exp(−*Ê*(*t*)/*N*)
m2	50 goats	Goats	**50**	Yes	1	1	0.038	1–exp(−*Ê*(*t*)/*N*)
m3	770 cows	**Cows**	770	Yes	1	1	**0.02**	1–exp(−*Ê*(*t*)/*N*)
m4	50 cows	**Cows**	**50**	Yes	1	1	**0.02**	1–exp(−*Ê*(*t*)/*N*)
Scenarios of sensitivity analysis								
s1	TimeIntro	Goats	770	Yes	**27**	**27**	0.038	1–exp(−*Ê*(*t*)/*N*)
s2	TimeBirth	Goats	770	Yes	1	**27**	0.038	1–exp(−*Ê*(*t*)/*N*)
s3	ProbAbUp	Goats	770	Yes	1	1	**0.38**	1–exp(−*Ê*(*t*)/*N*)
s4	ProbAbLow	Goats	770	Yes	1	1	**0.02**	1–exp(−*Ê*(*t*)/*N*)
s5	ProbInfUp	Goats	770	Yes	1	1	0.038	1–exp(−10*Ê(*t*)/*N*)
s6	NoYoung	Goats	770	**No**	1	1	0.038	1 – exp(−*Ê*(*t*)/*N*)

Overview of model parameters that have been varied in the 4 variations of the model and 6 scenarios of the sensitivity analysis. Bold font indicates a change relative to the default model of 770 goats.

In scenarios one and two of the sensitivity analysis, the moment of introduction of infection was varied. In both the original cow model as well as the default goat model, the simulations start with the introduction of an infected goat that just kidded (*t* = 1), initiating the transmission process. However, in the goat model the moment of introduction of an infected animal is set as the first day of the mating season, rather than at a random time during the year. In this way each simulation year started at the first day of the mating season and included a complete reproductive season, eliminating a possible source of variation, in that the timing of the introduction of an infected animal may influence the likelihood that the infection catches on in the population and the subsequent infection dynamics. To learn more about the effects of timing, in the first scenario both the introduction as well as the parturition of the infected animal were timed in the middle of the 12-week kidding season (at *t* = 27, six months after the start of the simulation). In this scenario the infection was not present in the herd during the six months between *t* = 1 and *t* = 27. In scenario two, the moment of introduction of the infected animal was kept at *t* = 1, but the parturition of the introduced infected animal was at *t*=27, unless by chance abortion due to *C. burnetii* occurred before that time.

In scenarios three and four, the probability of abortion due to Q fever (after a transition *S* to *I*_*1*_, *C*_*1*_ to *I*_*2*_ and *C*_*2*_ to *I*_*2*_) was adjusted. In scenario three, the abortion rate increased 10 fold, from 0.038 to 0.38, as it has been suggested in the literature that the abortion rate in individual goats may be higher than in cows [16]. In scenario four, the abortion rate decreased to 0.02, to exactly match the abortion rate in the cattle models. This scenario was used to investigate the sensitivity of the model to the scaling of the abortion rate that was applied due to the short gestation period of goats.

In scenario five, the probability *p* of infection (transition from *S* to *I*_*1*_ or from *C*_*2*_ to *I*_*2*_) was based on 10x the environmental load (instead of 1× the environmental load), according to 1 – exp(−10*Ê(*t*)/*N*). With this scenario, the sensitivity of the model was tested with regards to the scaling of the transmission rate. It can also be viewed as a potential scenario for currently unknown differences in the infection rate or in bacterial shedding levels in goats versus cows, since some differences in the relative proportion of each shedding route in the whole bacterial shedding have been reported [16].

In scenario six, youngstock were excluded from the model. The total herd size and age dependent culling rates remained unchanged. As in the cattle model, every year 282 uninfected goats that just kidded for the first time were introduced into the herd at random uniform times during the kidding season. With this scenario, the sensitivity to the addition of youngstock in the model was assessed.

### Model outputs

The four main outputs of interest of the model were, in short, (i) abortion patterns, expressed as annual incidence of abortions and rolling monthly incidence of abortions, (ii) environmental bacterial load, (iii) extinction of the infection, and (iv) the prevalence of shedders. A detailed description of these model outputs and the relation to the “real world” is given below.

From 2005–2009, unusually high numbers of abortions were observed in 28 commercial dairy goat herds in the Netherlands [9]. On 12 June 2008, abortion problems due to Q fever in dairy goat and dairy sheep herds in the Netherlands became notifiable. Abortion problems were defined as abortions in at least 5% of pregnant animals in large herds or in more than 3 animals in small herds (< 100 animals) within a period of 30 days [5]. In cattle, abortion storms featuring a high incidence of abortions during a short period of time have not been described. To investigate whether the difference in abortion patterns between cattle herds and dairy goat herds can be explained by differences in demographics, two measures for the abortion dynamics were computed: the annual incidence of abortions and a rolling monthly incidence of abortions. The annual incidence of abortions was defined as the annual cumulative number of abortions divided by the average herd size over a year, with the assumption that on average all animals in the herd have one pregnancy each year. The rolling monthly incidence of abortions was defined as the number of abortions that occurred in the herd over a four-week period divided by the number of animals that were pregnant at the start of this four-week period. Because the model has time-steps of one week, 52 rolling monthly incidences were computed for each model year, with the exception of the first four weeks of the simulation period.

Seasonal peaks of acute human Q fever infection were observed in the Netherlands during 2007, 2008 and 2009. The incidence of human cases was the highest from late spring to early summer, following the seasonal peaks of kidding in dairy goat herds. Furthermore, the incidence of human cases was the highest in geographic areas with high densities of dairy goat farms, the epidemic genotype among humans was also dominant among goats [8], and *C. burnetii* DNA was detected in inhalable size dust fractions within and around infected dairy goat farms [21]. All together this suggests that environmental contamination could form a link between bacteria shed by individual dairy goats within farms and human exposure to these bacteria. Therefore, the within-farm environmental bacterial load (model compartment *E*) was considered a main output of interest of the model. For each simulation, the area under the curve (AUC) of the environmental bacterial load was calculated over the 10 year simulation period by summing the environmental bacterial load at each time step (*t* = 0 until *t* = 520). For comparison between the different versions of the model, these AUC were expressed as a percentage of the mean AUC of the default model (770 goats), which was set to 100%.

Extinction of infection in goat herds versus cattle herds, relevant for the design of control measures, risk analyses and the formulation of policies are defined as the permanent absence of animals in states *I*_*1*_*, I*_*2*_*, I*_*3*_ and *C*_*1*_ for a period of at least 1 year. The mean time to extinction was calculated over all herds in which the infection had gone extinct.

The prevalence of shedders, which provides additional information relevant to the dynamics of abortions, environmental bacterial load, and extinction of the infection, was defined as the number of animals in *I*_*1*_ + *I*_*2*_ +*I*_*3*_ divided by the total number of animals in the herd, and was calculated for each time step in the model.

All analyses were performed using R software [22].

## Results

### Results of the comparison of herds

#### Abortion patterns

**Figure 2:**
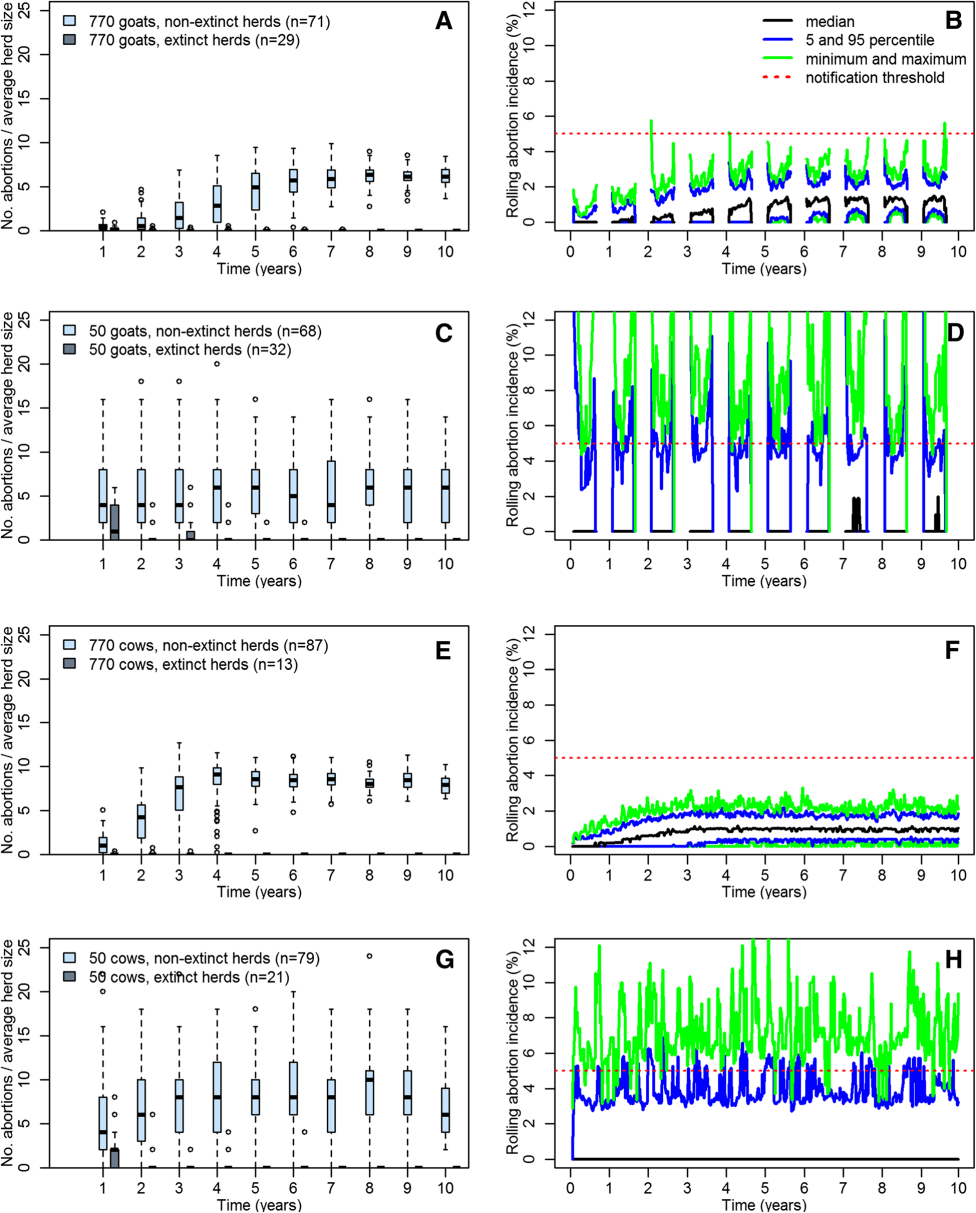
**Abortion patterns in the four models.** Boxplots for the annual incidence of abortions (defined as the annual number of abortions divided by the average herd size) in herds with- and without extinction of the infection after 10 years simulation (**A**, **C**, **E** and **G**) and rolling monthly incidence of abortions (defined as the number of abortions over four weeks divided by the number of animals pregnant at start of a four week period) in herds without extinction (**B**, **D**, **F** and **H**). The red dotted line in the panels **B**, **D**, **F** and **H** indicate the 5% rolling monthly abortion incidence that was notifiable in the Netherlands during the epidemic. In the legends, the numbers in between brackets indicate the total number of herds which got rid of the infection over the 10 years of simulation, and the number of herds which did not. **A, B)** model with 770 goats; **C, D)** model with 50 goats; **E, F)** model with 770 cows; **G, H)** model with 50 cows.

The annual abortion incidence in the default model with 770 goats increased gradually up to a steady state of ~ 6 abortions per 100 animals per year. In the model with 770 cows, the steady state incidence was ~ 8 abortions per 100 animals per year and steady state was reached sooner, as can be seen in Figure 2. In the herds with 50 animals, the mean annual abortion incidence during the tenth simulation year was ~6% in goat herds, and ~7% in cattle herds. During simulations, as long as the annual abortion incidence had not exceeded 1%, the infection could still go extinct in herds with 770 animals. In herds of 50 animals, extinction of the infection could still occur after higher annual abortion incidences: up to 6% in goats and up to 8% in cattle. The variation in abortion incidence was larger in small herds than in large herds, and a steady state was reached faster in small herds than in large herds. The annual abortion incidences in the four models are displayed in Table 3 and Figure 2.

**Table 3:** Overview of model outputs

**Model**	**Model code**	**Abortion incidence**		**Environmental bacterial load**		**Extinction rate (%)**	**Time to extinction (weeks)**		**Prevalence of shedders (%)**	
		**mean**	**median,**	**mean**	**median,**		**mean**	**median,**	**mean**	**median,**
			**5-95%**		**5-95%**			**5-95%**		**5-95%**
Variations of the model for goat and cow farm infection										
m1	770 goats	6.2	6.1, 4.4–8.1	100	99, 53–142	29	145	111, 18–296	30	30, 25–34
m2	50 goats	5.6	6.0, 0–12	10	10, 4–14	32	162	144, 21–366	26	26, 7–45
m3	770 cows	8.0	7.9, 6.6–9.8	269	277, 184–320	13	67	51, 16–156	44	45, 40–48
m4	50 cows	7.1	6.0, 2.0–14	21	20, 14–28	21	102	72, 27–225	43	42, 28–57
Scenarios of sensitivity analysis of goat farm infection										
s1	TimeIntro	6.1	6.4, 4.4–8.1	83	90, 40–119	40	102	99, 1–280	29	30, 23–34
s2	TimeBirth	6.2	6.2, 4.2–8.5	82	88, 23–116	50	74	37, 5–219	29	30, 24–34
s3	ProbAbUp	24.8	24.8, 22–27.3	229	231, 209–246	4	25	24, 7–44	33	33, 29–36
s4	ProbAbLow	3.5	3.5, 2.4–4.7	82	85, 22–126	34	128	106, 21–317	28	29, 21–34
s5	ProbInfUp	8.3	8.3, 6.8–10.1	267	267, 255–282	1	1	1	52	52, 49–55
s6	NoYoung	6.4	6.4, 3.6–8.3	86	92, 18–129	34	172	162, 42–333	28	29, 15–35

Abortion incidence: incidence of abortions during the 10^th^ simulation year in non-extinct herds; Environmental load AUC: area under the curve of the environmental load over 10 years simulation in non-extinct herds, expressed as percentage of the mean AUC of model m1; Ext. rate (%): percentage of herds with extinction of infection after 10 years simulation; Time to extinction: the number of weeks from introduction of an infected animal in the herd until extinction of the infection; Prevalence of shedders: prevalence of shedders at t = 520 in non-extinct herds.

The rolling four-week abortion incidence in the model with 770 goats in nearly all cases did not exceed the 5% incidence that was notifiable in the Netherlands during the epidemic. The notification threshold was exceeded slightly in a few herds at the start or end of the kidding season, when only a limited number of animals were at risk to abort. In the herds with 770 cows, the rolling incidence stayed well below 5%. In small goat and cattle herds, the rolling abortion incidence was very variable, and rolling incidences higher than 5% were regularly observed. The four-week rolling abortion incidences of the four models are displayed in Figure 2. The four-week rolling abortion incidences of four individual simulations of the model with 770 goats are provided in Additional file 2.

#### Environmental bacterial load

**Figure 3:**
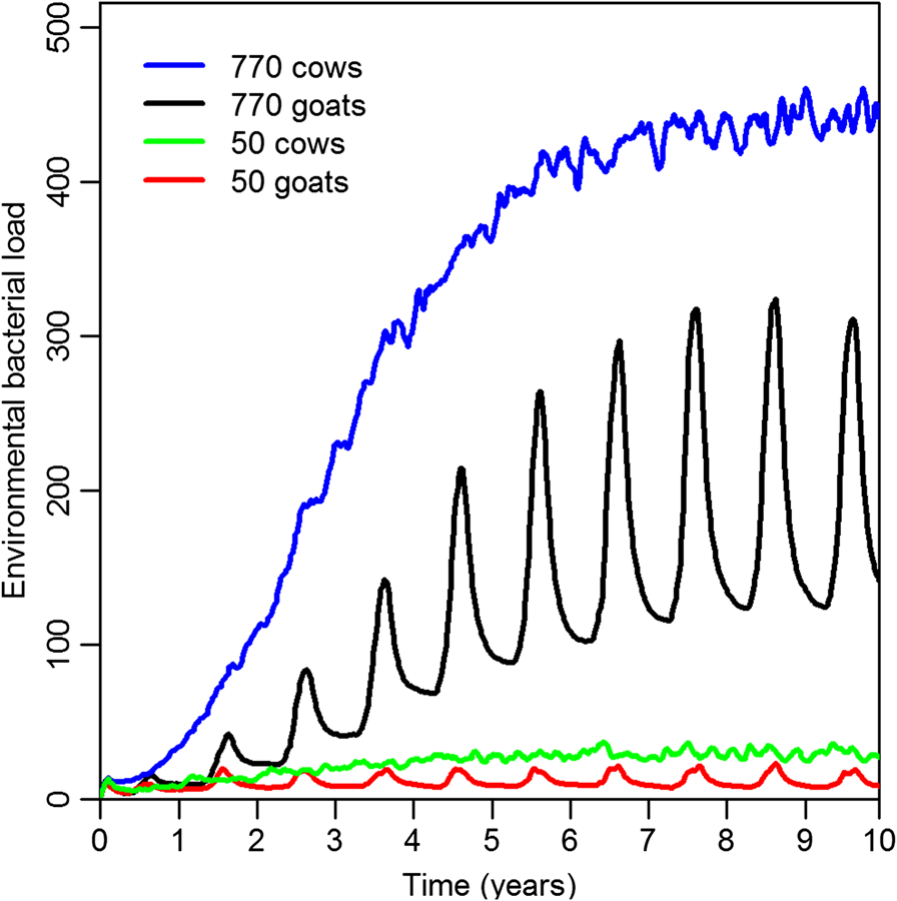
**Environmental bacterial load.** Temporal dynamics of the mean environmental bacterial load in the four models.

The environmental bacterial load AUC over a ten year simulation in non-extinct herds was larger in large herds than in small herds. When comparing herds of the same size, the environmental load was larger in cattle herds than in goat herds. In the goat herds, the environmental load showed seasonal peaks, while the environmental load in the cattle herds was relatively constant over time. In all modeled herds, the environmental bacterial load increased gradually over time until it reached a steady state, which was achieved more quickly in small herds than in large ones. The mean environmental bacterial load in the four models is presented in Figure 3.

The mean AUC after 10 years simulation of herds with 770 goats without extinction of the infection was set to 100%. For the other three models, the mean AUC relative to the default model AUC were 10% for non-extinct herds with 50 goats, 269% for 770 cows, and 21% for 50 cows (Table 3).

#### Extinction of the infection

**Figure 4:**
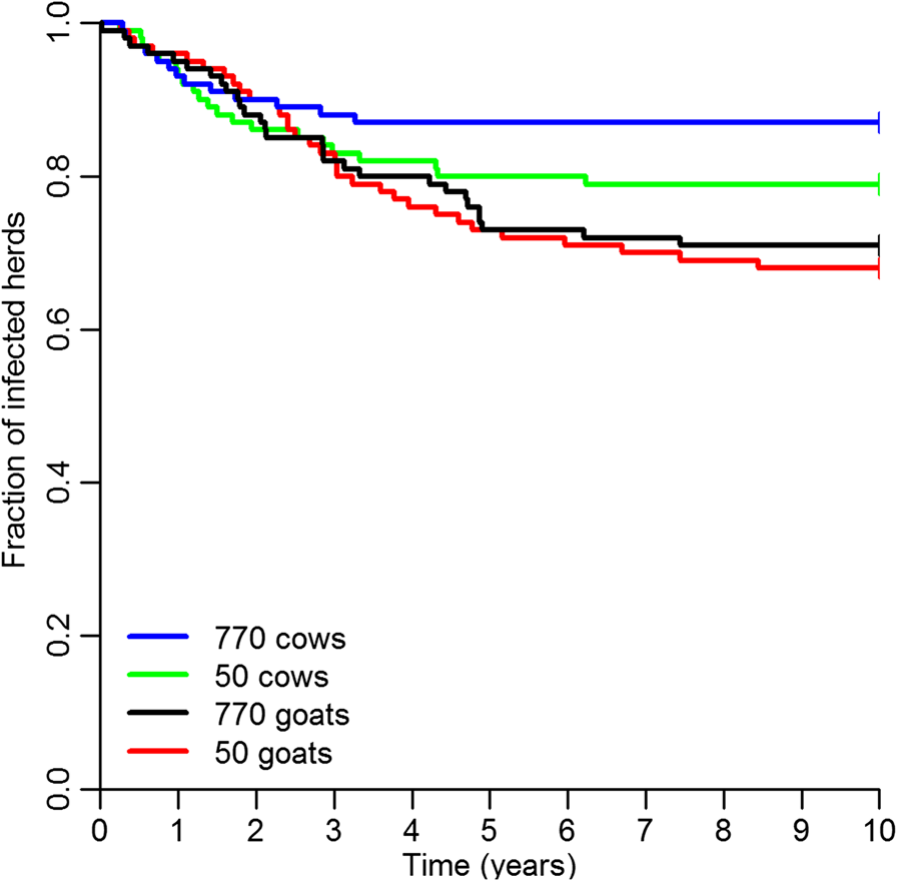
**Extinction of infection.** Kaplan Meier curve for extinction of infection in the four models.

Extinction of the infection occurred more often in small cattle herds than in large cattle herds. When comparing herds of the same size, extinction was more common in goat herds than in cattle herds. After 10 years simulation, the infection was extinct in 29% of herds with 770 goats, 32% of herds with 50 goats, 13% of herds with 770 cows, and in 21% of herds with 50 cows (Table 3). The mean time to extinction was 145 weeks in herds with 770 goats, 162 weeks in herds with 50 goats, 67 weeks in herds with 770 cows, and 102 weeks in herds with 50 cows (Table 3). The dynamics of extinction and persistence of the infection in the four models are shown in Figure 4.

#### Prevalence of shedders

**Figure 5:**
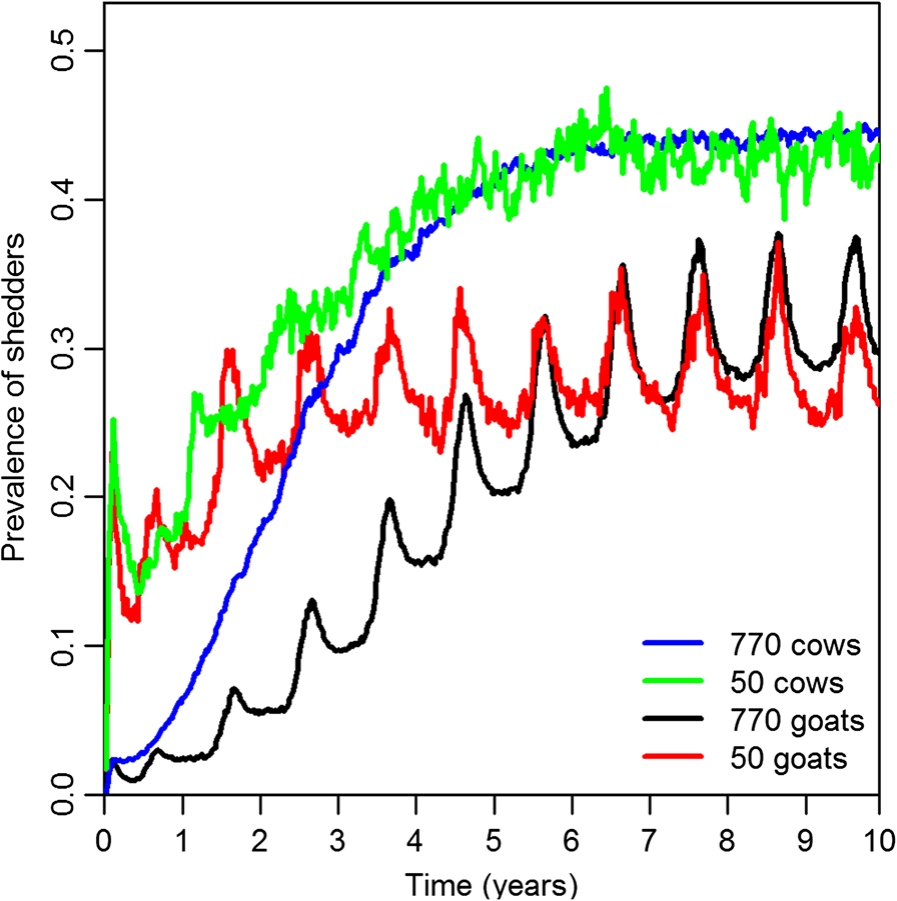
**Prevalence of shedders.** Temporal dynamics of the mean prevalence of shedders in the four models.

The prevalence of shedders in non-extinct herds increased faster in small herds than in large herds. In the steady state, the prevalence of shedders in goat herds showed seasonal fluctuations, while the prevalence in cattle herds stayed relatively constant. Overall, the prevalence of shedders was higher in cattle herds than in goat herds. At the last day of the simulation period (*t* = 520), the mean prevalence of shedders was 30% in herds with 770 goats, 26% in herds with 50 goats, 44% in herds with 770 cows, and 43% in herds with 50 cows, see Figure 5.

### Results of the sensitivity analyses

Annual and rolling abortion incidences, environmental load dynamics, extinction curves and prevalence dynamics of all sensitivity analyses are shown in Additional files 3, 4, 5, 6 and 7.

#### Sensitivity to changes in timing of introduction of infection into the herd

Introduction of infection in the middle of the kidding season (scenario 1 and 2 of the sensitivity analysis) instead of at the start of the mating season (default model) increased the likelihood of extinction of the infection. The likelihood of extinction was 29% in the default model, 40% when the infected animal was introduced and kidded at *t* = 27 (scenario 1), and 50% when the infected animal was introduced at *t* = 1 but kidded at *t* = 27 (scenario 2), see Table 3 and Additional file 3. Once a steady state of infection was reached, the rolling abortion incidences in scenarios one and two were similar to the default model (Additional file 4).

#### Sensitivity to changes in the probability of abortion

When the probability of abortion was increased tenfold (scenario 3), the likelihood of extinction decreased from 29% to 4%. The annual incidence of abortions was increased, peaking in the second year, with a mean of 45 and a maximum of 62 abortions per 100 animals. After this peak, the annual abortion incidence decreased to a steady mean incidence of ~25% per year (Table 3 and Additional file 5). The mean four-week rolling abortion incidence was well above the 5% “notifiable” incidence during the first two kidding seasons, and remained little over 5% during the remaining kidding seasons. In individual simulations, peaks up to ~40% were observed in the four-week rolling abortions incidence. High peak incidences were usually followed by a steep decline the following season (Additional file 2). The mean environmental bacterial load peaked during the first two years, and then decreased to a steady state well above the steady state of the default model (Additional file 6).

When the probability of abortion was decreased (scenario 4), the annual abortion incidence and the rolling abortion incidence increased very gradually over the years, up to a mean annual abortion incidence of 3.5% in the 10th year (Table 3 and Additional file 5).

#### Sensitivity to an increased probability of infection

When the infection rate was increased tenfold (scenario 5), the likelihood of extinction dropped to 1%. The prevalence of shedders was higher in this scenario than in all other model variants. It peaked up to a mean of 59% in the first year, and then slowly decreased to a mean of 52% after 10 years (Table 3 and Additional file 7). The annual incidence of abortions was ~ 8% during the entire simulation period (Additional file 5). The environmental bacterial load peaked in the second year (although not as high as in scenario 3) and fluctuated at a high level for the remaining years (Additional file 6).

#### Sensitivity to no youngstock in the herd

In the model without youngstock (scenario 6), the mean annual abortion incidence as well as the likelihood of extinction were fairly similar to the default model. However, the seasonally peaked nature of the environmental bacterial load and the prevalence of shedders was less pronounced than in the default model. Between the kidding seasons, the environmental bacterial load and prevalence of shedders were similar to the default model, while during kidding seasons, the peaks were lower than in the default model.

## Discussion

In this study, we explored whether the emergence of Q fever in goats in the Netherlands could be explained by demographic characteristics of large intensive dairy goat husbandry systems versus the demographics of small cattle herds where no large epidemics have been observed. Using a modeling approach, it was found that the occurrence of “abortion storms” and the heterogeneity of the abortion patterns across herds in intensive goat husbandry systems in the Netherlands could not be explained by the demographics of Dutch dairy goat herds alone, and most likely aspects of the manifestation of the infection in the host are essential in explaining the observed abortion patterns.

The annual incidence of abortions and the environmental bacterial load were higher in cattle herds than in goat herds of the same size, and the infection was more likely to go extinct in goat herds than in cattle herds. In small herds (50 animals), the likelihood of extinction of the infection was greater than in larger herds (770 animals) of the same species. Provided that infection persisted, a steady state of infection dynamics was reached faster in small herds than in large herds.

In the model with 770 goats, which represented the average Dutch dairy goat herd, the abortion patterns seemed to reproduce the patterns observed in the *majority* of Dutch infected herds fairly well. As in real life, the abortions in the model occurred in waves due to the seasonal reproduction patterns, and as in most infected Dutch dairy goat herds, the modeled rolling abortion incidence over 4 weeks did not exceed the 5% level that was notifiable in the Netherlands during the time of the epidemic. The annual abortion incidence of ~ 6% in the steady state seemed a bit high because such repeated abortion waves have not been reported, but it may be within reasonable limits for endemically infected herds. The “abortion storms” with high abortion incidences observed within some herds and the large variability in abortion patterns observed across herds were not reproduced.

In the Netherlands 94 herds were classified as infected between 2005 and mid-2010, and abortion problems were reported in only 28 herds, with abortion incidences that range from just over the notification threshold of 5% up to 80% in subgroups of herds [9,23]. The fact that abortion storms were rare suggests a possible role of stochasticity related to seasonal kidding. Indeed, in the simulated goat herds the rolling abortion incidence has higher peaks than in cattle herds of the same size (Figure 2, upper green lines). However, the fact that goat herds are much bigger than cattle herds results in lower rolling abortion incidences since this reduces the effect of stochasticity (Figure 2). One explanation for the abortion storms observed in the field could be that the environmental contamination is not equally distributed over entire herds, but is clustered in subgroups within a herd. In that case the model with fewer animals may be more representative, and indeed the model with 50 goats shows higher variability in the infection dynamics across herds and rolling abortion incidences above the notification threshold.

An alternative explanation for the absence of abortion storms in the model is related to the result that the environmental load and prevalence of shedders was lower in goat than in cattle herds. The kidding pattern of dairy goats appears to be the major driver of this difference. Due to the seasonality and short gestation period of goats, pregnant animals were not present in the goat herds during 19 weeks of the year, and therefore abortions with associated shedding of large quantities of bacteria could not occur during those periods. Hence, the environmental bacterial load gradually decreased after each kidding season, and increased again during the following kidding season. As observed in the simulations, in cattle herds, the continuous presence of animals at risk to abort kept the infection running at a higher level than on seasonally kidding goat herds. In addition, in the model it was assumed that all animals get pregnant every year, while farmers commonly choose for fewer pregnancies per goat, for instance once every two years, as goats are capable of extended lactation without a new delivery. The presence of fewer pregnant animals in the herd would result in even lower infection levels.

In addition to the kidding pattern and herd size, also the presence of youngstock in goat herds influences the within-herd infection dynamics. In herds without youngstock (scenario 6), a higher turnover of animals was required to keep the average herd size at 770 animals. This meant that without youngstock, more potentially infected animals were removed, and more susceptible animals were introduced annually, which resulted in a ‘wash-out’ of infected animals and thereby lower infection levels. Indeed, in goat herds with youngstock (default model) the prevalence of shedders has higher peaks than in goat herds without youngstock (Additional file 7). Thus, the presence of youngstock increases prevalence, but not sufficiently to explain the high rolling abortion incidences observed in the field.

Thus, one of the main conclusions from our model is that the “abortion storms” observed in intensive goat husbandry systems in the Netherlands are not wholly explained by the herd size, reproductive pattern, presence of youngstock and other demographic characteristics of dairy goats in the intensified husbandry system in the Netherlands. Therefore it is most likely that aspects of the manifestation of the infection in the host are essential in explaining the observed abortion storms.

One possibility may be that the model itself inaccurately describes some crucial part of the infection, even though it was constructed based on longitudinal field observations on 5 cattle herds and the simulated infection dynamics are consistent with field data from dairy cattle herds in western France [12]. For example, the model assumed that animals have a risk to abort during three weeks following each event of infection, reinfection or resumption of shedding (i.e. *S*–*I*_*1*_*, C*_*1*_–*I*_*2*_ and *C*_*2*_–*I*_*2*_ transitions) [15]. In reality this may be different, e.g. abortions may occur much later or only after initial infection, and although resumption of shedding is likely to follow abortion, it may not necessarily lead to abortion. Further, the gestation status (pregnant or non-pregnant) and phase of gestation (e.g. early or late) at the time of infection may influence the resulting manifestation of the infection (e.g. probability of abortion, immunological response, shedding pattern). The potential effect of timing of infection relative to gestation on the manifestation of the infection may play out differently at the herd level in herds with synchronized reproduction cycles (i.e. goat herds) versus herds with year-round pregnancies and deliveries (cattle herds). Data on gestation-stage dependent Q-fever infection dynamics in ruminants are sparse, and therefore these dynamics were not included. If the reason for the absence of abortion storms is indeed in the model structure itself, it is not clear whether this points at a fundamental difference between cows and goats, or whether the same aspects of the model structure are inaccurate for cattle as well.

A second possibility is that the infection process in the individual animals (i.e. the parameter values of the epidemiological part of the model) is different in goats than in cattle. In the model, the abortion and infection rates were scaled based on assumptions of similarity. When the probability of abortion was increased 10-fold in the model, this resulted in a peak in the mean annual abortion incidence during the second year after introduction of the infection, followed by a decrease to a steady 25% mean annual abortion incidence. In some herds the four-week rolling abortion incidences peaked up to ~40% followed by a steep decline the following season (Additional file 2), more resembling real herds. When the infection rate was increased 10-fold, this also resulted in more abortions (Additional files 4 and 5).

If the abortion rate or infection rate is indeed different between cattle and goats, several mechanisms might explain this difference. From the model it cannot be concluded whether such mechanisms are pathogen-related (e.g. different strains, evolution of virulence), host-related (e.g. host susceptibility, immunological response), within-herd environment related (e.g. barn characteristics) or a combination. For instance, available phylogenetic evidence suggests that the Dutch epidemic was caused by one dominant strain [8,9], which may have differed in terms of abortion or infection rate from the strain circulating in the cattle herds used to parameterize the model. Host related explanations include that goats may be more sensitive to abort due to Q fever than cattle, that the Dutch dairy goat herds may have been fully immunologically naïve (unlike the endemically infected cattle herds used to parameterize the model), or that goats show a difference in their bacterial shedding pattern or shedding levels during parturition and abortion that led to more environmental contamination and thus higher infection rates [16]. Environmental explanations include that the infection rate may be higher in goats due to the density of animals in the barn or to the deep litter boxes in which goats are kept.

In conclusion, the abortion storms in Dutch dairy goat herds could not be fully explained by demographics alone. Although most likely aspects of the manifestation of the infection in the host are essential in explaining the observed abortion patterns, there are gaps in the current understanding of the drivers of Q fever abortion storms, and this may hamper the prevention of future problems on goat farms and in humans living close to goat farms. Our finding that cattle herds may have higher levels of within-herd environmental bacterial load than goat herds after introduction of infection into a fully susceptible herd may be taken into account when enlarging cattle farming systems.

## Competing interests

The authors declare that they have no competing interests.

## Authors’ contributions

LH participated in the design of the study, carried out the model adjustments and analyses, and drafted the manuscript. AC, FB and EV participated in the design of the study, and helped to draft the manuscript. DK and MN participated in the design and coordination of the study and helped to draft the manuscript. All authors read and approved the final manuscript.

## Supplementary Material

Additional file 1 **Parameter definitions and values for the epidemiological model.** Table with definitions of the epidemiological model parameters and their values used for simulations [13,24,25]. Click here for file

Additional file 2 **Rolling abortion incidence for individual simulations.** Figure of the rolling monthly incidence of abortions in 4 individual simulations for the model with 770 goats and 4 individual simulations of the sensitivity analysis for an increased probability of abortion. Rolling monthly incidence of abortions (defined as the number of abortions over four weeks divided by the number of animals pregnant at start of four week period) in 4 individual simulations of the model with 770 goats (**A – D**) and 4 individual simulations of the sensitivity analysis for an increased probability of abortion (**E – H**). The red dotted lines indicate the 5% rolling monthly abortion incidence that was notifiable in the Netherlands during the epidemic. The individual runs were selected to display the most extreme peaks in rolling abortion incidence (A, E & F) as well as a variety of more common abortion incidence patterns (B, C, D, G & H). Click here for file

Additional file 3 **Extinction of infection in the sensitivity analyses.** Kaplan Meier curve for extinction of infection in the default model and six sensitivity analyses. Click here for file

Additional file 4 **Rolling abortion incidence in the sensitivity analyses.** Figure of the rolling monthly incidence of abortions for the 6 scenarios of the sensitivity analysis. Rolling monthly incidence of abortions (defined as the number of abortions over four weeks divided by the number of animals pregnant at start of four week period) in herds without extinction for the 6 scenarios of the sensitivity analysis. The red dotted lines indicate the 5% rolling monthly abortion incidence that was notifiable in the Netherlands during the epidemic. Click here for file

Additional file 5 **Annual abortion incidence in the sensitivity analyses.** Figure of the annual incidence of abortions for the 6 scenarios of the sensitivity analysis. Boxplots for the annual incidence of abortions (defined as the annual number of abortions divided by the average herd size) in herds with- and without extinction of the infection for the 6 scenarios of the sensitivity analysis. Click here for file

Additional file 6 **Environmental bacterial load in the sensitivity analyses.** Figure of the temporal dynamics of the mean environmental bacterial load in the default model and six sensitivity analyses. Click here for file

Additional file 7 **Prevalence of shedders in the sensitivity analyses.** Figure of the temporal dynamics of the mean prevalence of shedders in the default model and six sensitivity analyses. Click here for file
