# Supplementary material for: Dairy goat demography and Q fever infection dynamics
Source: Vet Res. 2013 Apr 26;44(1):28. doi: 10.1186/1297-9716-44-28 (PMC3648346; doi:10.1186/1297-9716-44-28)
Supplement: Additional file 1 — Parameter definitions and values for the epidemiological model. Table with definitions of the epidemiological model parameters and their values used for simulations [13,24,25]. [file 1297-9716-44-28-S1.docx]

# Additional file 1 Parameter definitions and values for the epidemiological model. This table is adapted from Courcoul et al. [15] and provides definitions of the epidemiological model parameters and their values used for simulations. Bold font indicates a change relative to the value in the original cow model.

| **Parameter** | | **Definition** | **Value in cow model** | **Value in goat model** | **Source** |
| --- | --- | --- | --- | --- | --- |
| *p* (week^-1^) | | Infection rate *S* => *I_1_* | 1 – exp(-E(t)) | **1 – exp(-Ê (t)/N)** | Courcoul et al. [13] |
| *m* (week^-1^) | | Transition rate *I_1_* => *S* | 0.7 | 0.7 |  |
| *q* (week^-1^) | | Transition rate *I_1_* => (*I_2_* or *I_3_*) | 0.02 | 0.02 |  |
| *r_2_* (week^-1^) | | Transition rate *I_3_* => *C_1_* | 0.02 | 0.02 | Assumption that *I_3_* shed 10 times longer than *I_2_* |
| *s* (week^-1^) | | Transition rate *C_1_* => *I_2_* | 0.15 | 0.15 | Courcoul et al. [13] |
| *τ* (week^-1^) | | Transition rate *C_1_* => *C_2_* | 0.0096 | 0.0096 | Based on Fournier et al. [24] and Plommet et al. [25], assumption that the mean life duration of antibodies in cattle is 2 years |
| *μ (*week^-1^) | | Decay rate of *C. burnetii* | 0.2 | 0.2 | Courcoul et al. [13] |
| *probav* | | Probability of abortion after a transition *S* => *I_1_*, *C_1_* => *I_2_* and *C_2_* => *I_2_* | 0.02 | **0.02*(40/21)** | Calibrated to match the distribution of abortions observed in cattle in the field (A.F. Taurel, 2010, personal communication) |
| *ρ^mf^* | | Proportion of bacteria shed through mucus/faeces filling the environmental compartment | 0.28 | 0.28 | Calibrated from expert opinion to match the environmental bacterial load inferred in Courcoul et al. [13] |
| ratio *ρ^milk^/ ρ^mf^* | | *ρ^milk^* = proportion of bacteria shed through milk filling the environmental compartment | 0.125 | 0.125 |  |
| *α* | milk | Probability distribution of the shedding routes for the *I_1_* individuals | 0.31 | 0.31 | From field data (R. Guatteo 2009, personal communication) |
|  | mucus/feces |  | 0.62 | 0.62 |  |
|  | milk+ mucus/feces |  | 0.07 | 0.07 |  |
| *β* | milk | Probability distribution of the shedding routes for the *I_2_* individuals after 4 weeks postpartum | 0.61 | 0.61 |  |
|  | mucus/feces |  | 0.33 | 0.33 |  |
|  | milk+ mucus/feces |  | 0.06 | 0.06 |  |
| *β_calv_* | milk | Probability distribution of the shedding routes for the *I_2_* individuals in the 4 first weeks postpartum | 0.14 | 0.14 |  |
|  | mucus/feces |  | 0.5 | 0.5 |  |
|  | milk+ mucus/feces |  | 0.36 | 0.36 |  |
| *γ* | milk | Probability distribution of the shedding routes for the *I_3_* individuals after 4 weeks postpartum | 0.83 | 0.83 |  |
|  | milk+ mucus/feces |  | 0.17 | 0.17 |  |
| *γ_calv_* | milk | Probability distribution of the shedding routes for the I_3_ individuals in the 4 first weeks postpartum | 0.25 | 0.25 |  |
|  | milk+ mucus/feces |  | 0.75 | 0.75 |  |
| *Q1* | low level | Probability distribution of the shedding levels for all the *I_1_* and for the *I_2_* shedding in mucus/faeces after 4 weeks postpartum | 0.85 | 0.85 |  |
|  | mid level |  | 0.15 | 0.15 |  |
|  | high level |  | 0 | 0 |  |
| *Q2* | low level | Probability distribution of the shedding levels for the *I_2_* shedding in milk after 4 weeks postpartum | 0.4 | 0.4 |  |
|  | mid level |  | 0.5 | 0.5 |  |
|  | high level |  | 0.1 | 0.1 |  |
| *Q3* | low level | Probability distribution of the shedding levels for all the *I_2_* in the 4 first weeks postpartum | 0.2 | 0.2 |  |
|  | mid level |  | 0.25 | 0.25 |  |
|  | high level |  | 0.5 | 0.5 |  |
| *Q4* | low level | Probability distribution of the shedding levels for the *I_3_* shedding in mucus/faeces after 4 weeks postpartum | 0.6 | 0.6 |  |
|  | mid level |  | 0.4 | 0.4 |  |
|  | high level |  | 0 | 0 |  |
| *Q5* | low level | Probability distribution of the shedding levels for all the *I_3_* shedding in milk and for the *I_3_* shedding in mucus/faeces in the 4 first weeks postpartum | 0.15 | 0.15 |  |
|  | mid level |  | 0.6 | 0.6 |  |
|  | high level |  | 0.25 | 0.25 |  |
| *Qty* (units of environment) | low level | Quantity of bacteria released by shedders in low, mid and high levels respectively, expressed in dimensionless units. | 1/3000 | **(1/3000)*50** | Ratio between the 3 levels calculated from field data (R. Guatteo 2009, personal communication) |
|  | mid level |  | 1/30 | **(1/30)*50** |  |
|  | high level |  | 1 | **1*50** |  |
