# Supplementary figures and images for: Dairy goat demography and Q fever infection dynamics
Source: Vet Res. 2013 Apr 26;44(1):28. doi: 10.1186/1297-9716-44-28 (PMC3648346; doi:10.1186/1297-9716-44-28)

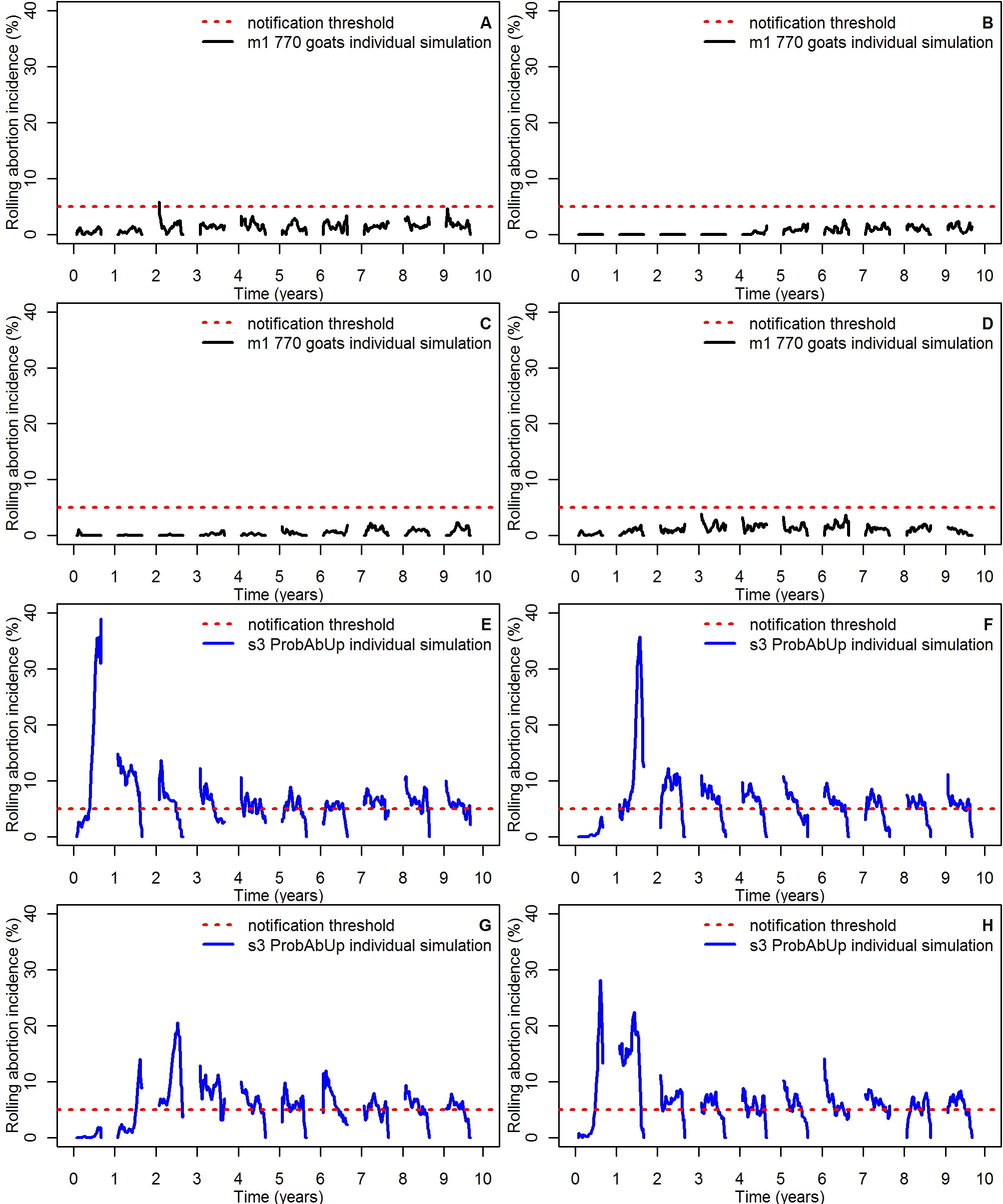


**Additional file 2 Rolling abortion incidence for individual simulations.**

Supplement: Additional file 2 — Rolling abortion incidence for individual simulations. Figure of the rolling monthly incidence of abortions in 4 individual simulations for the model with 770 goats and 4 individual simulations of the sensitivity analysis for an increased probability of abortion. Rolling monthly incidence of abortions (defined as the number of abortions over four weeks divided by the number of animals pregnant at start of four week period) in 4 individual simulations of the model with 770 goats (A – D) and 4 individual simulations of the sensitivity analysis for an increased probability of abortion (E – H). The red dotted lines indicate the 5% rolling monthly abortion incidence that was notifiable in the Netherlands during the epidemic. The individual runs were selected to display the most extreme peaks in rolling abortion incidence (A, E & F) as well as a variety of more common abortion incidence patterns (B, C, D, G & H). [file 1297-9716-44-28-S2.docx]

**Additional file 3 Extinction of infection in the sensitivity analyses.**


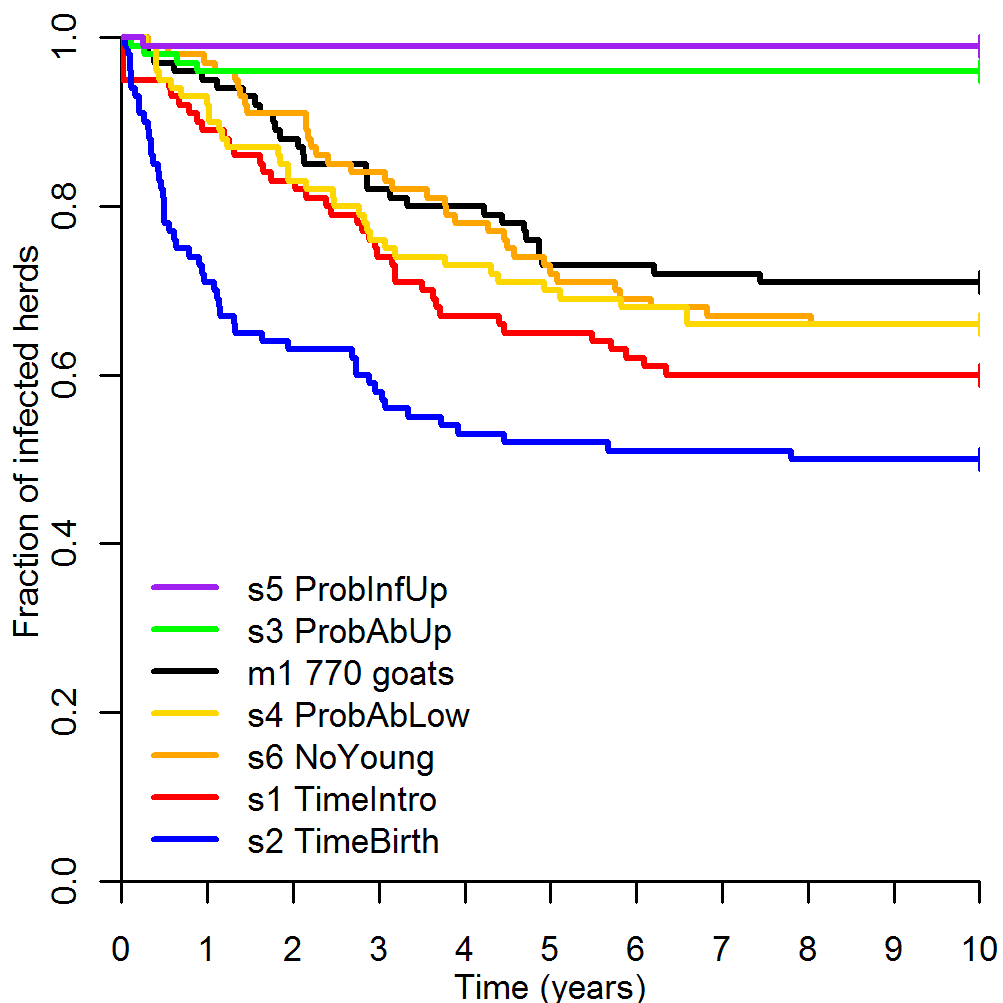

Supplement: Additional file 3 — Extinction of infection in the sensitivity analyses. Kaplan Meier curve for extinction of infection in the default model and six sensitivity analyses. [file 1297-9716-44-28-S3.docx]

***
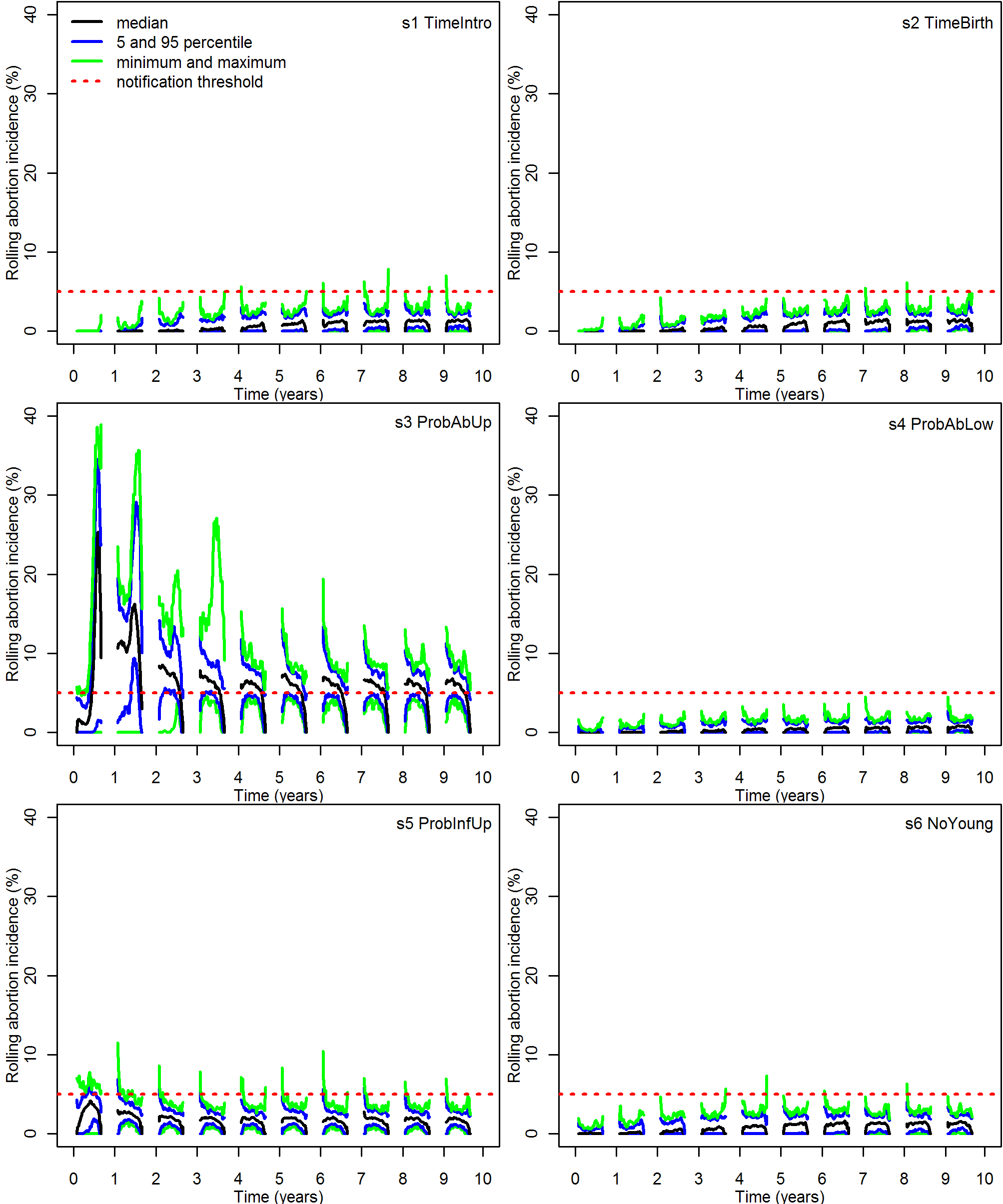
***

**Additional file 4 Rolling abortion incidence in the sensitivity analyses.**

Supplement: Additional file 4 — Rolling abortion incidence in the sensitivity analyses. Figure of the rolling monthly incidence of abortions for the 6 scenarios of the sensitivity analysis. Rolling monthly incidence of abortions (defined as the number of abortions over four weeks divided by the number of animals pregnant at start of four week period) in herds without extinction for the 6 scenarios of the sensitivity analysis. The red dotted lines indicate the 5% rolling monthly abortion incidence that was notifiable in the Netherlands during the epidemic. [file 1297-9716-44-28-S4.docx]

***
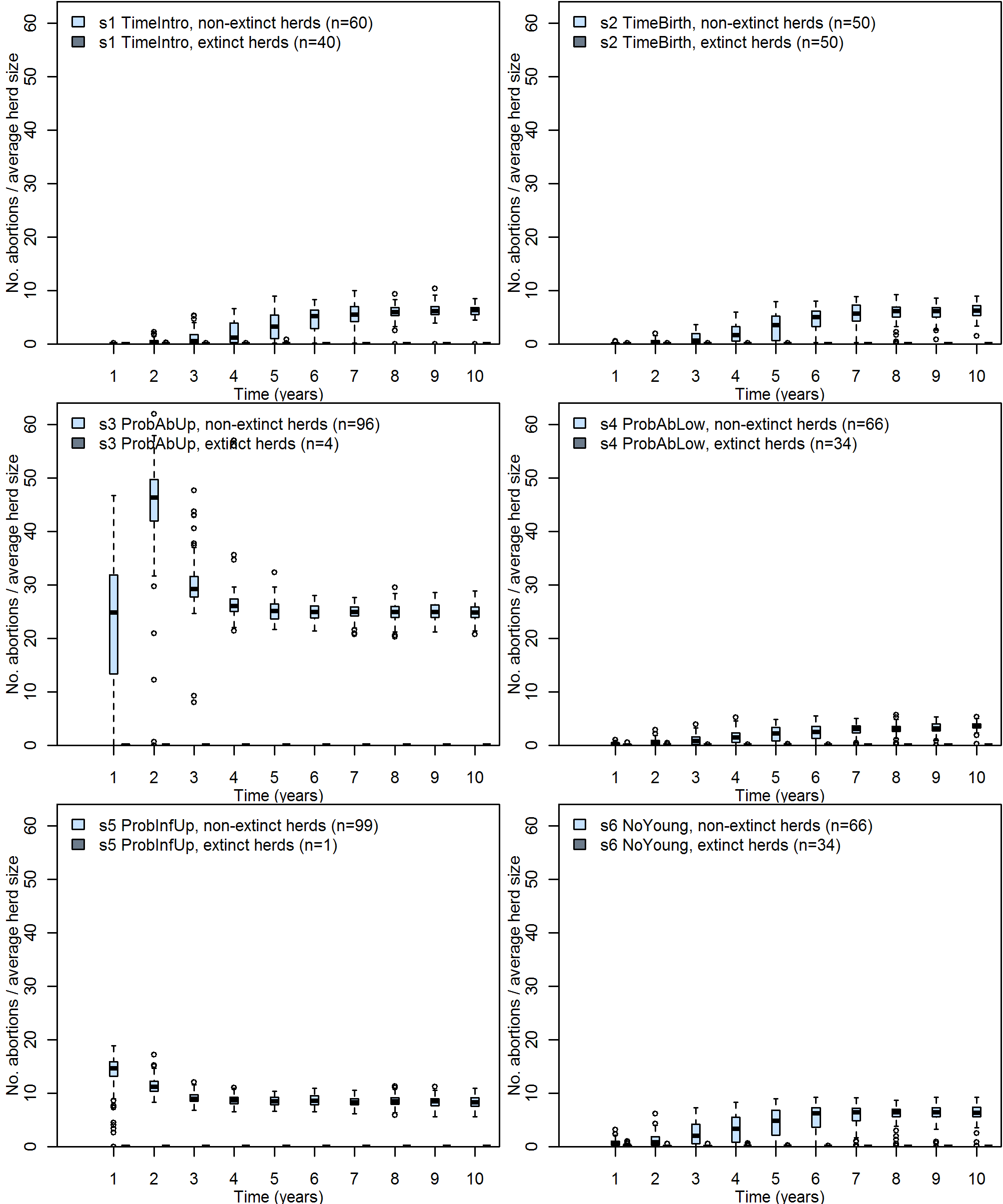
***

**Additional file 5 Annual abortion incidence in the sensitivity analyses.**

Supplement: Additional file 5 — Annual abortion incidence in the sensitivity analyses. Figure of the annual incidence of abortions for the 6 scenarios of the sensitivity analysis. Boxplots for the annual incidence of abortions (defined as the annual number of abortions divided by the average herd size) in herds with- and without extinction of the infection for the 6 scenarios of the sensitivity analysis. [file 1297-9716-44-28-S5.docx]

**Additional file 6 Environmental bacterial load in the sensitivity analyses.**


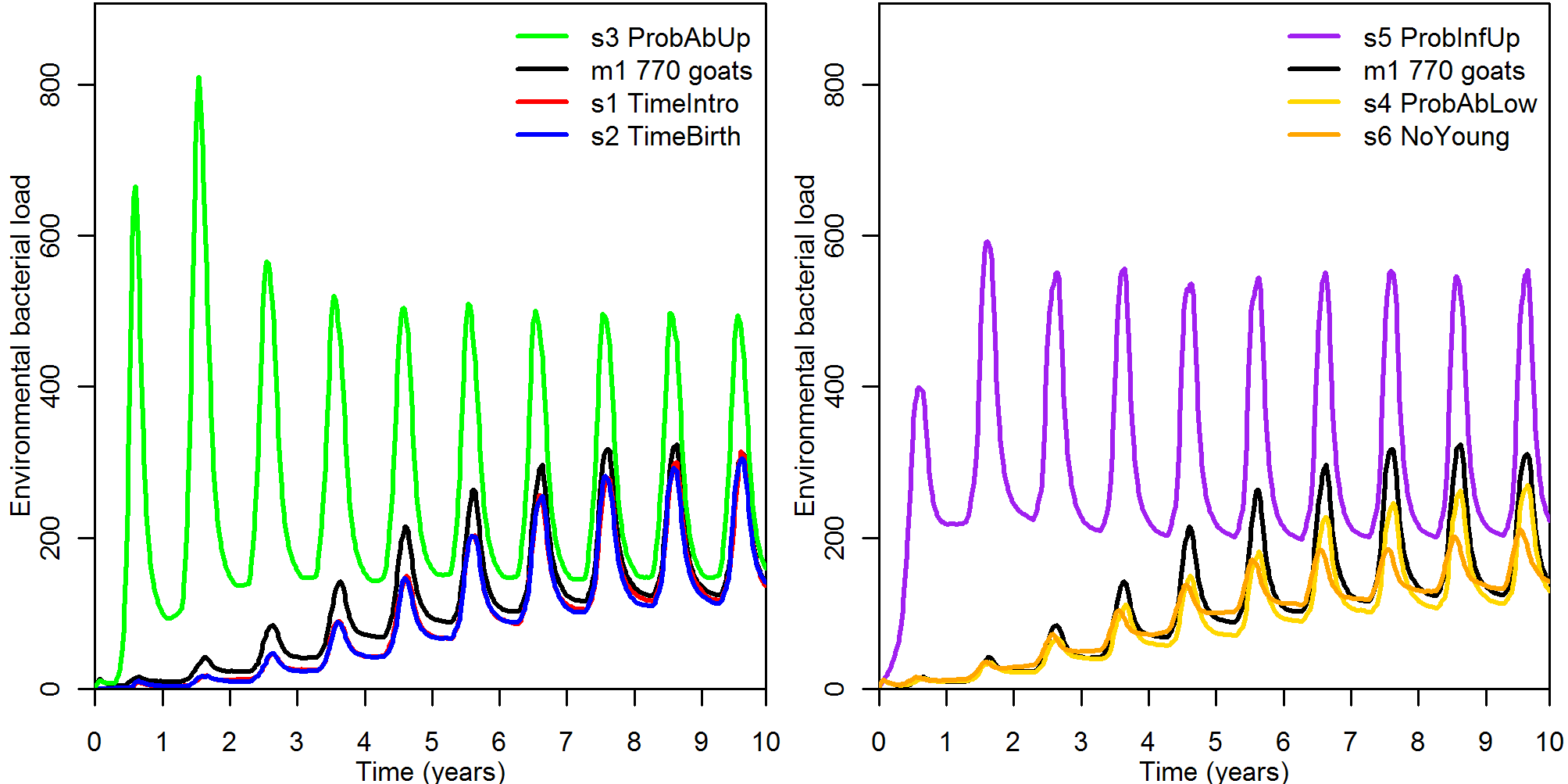

Supplement: Additional file 6 — Environmental bacterial load in the sensitivity analyses. Figure of the temporal dynamics of the mean environmental bacterial load in the default model and six sensitivity analyses. [file 1297-9716-44-28-S6.docx]

**Additional file 7 Prevalence of shedders in the sensitivity analyses.**


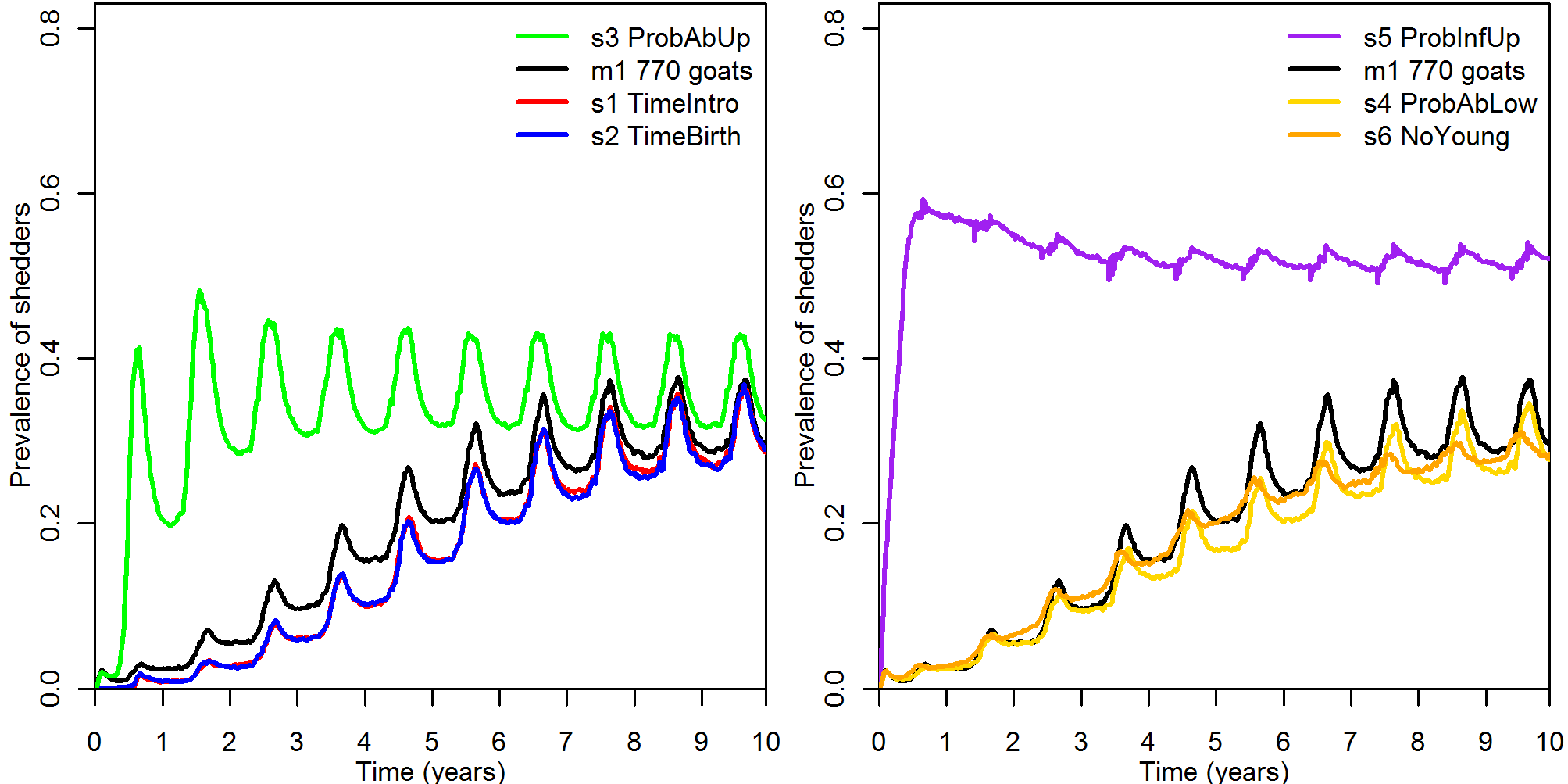

Supplement: Additional file 7 — Prevalence of shedders in the sensitivity analyses. Figure of the temporal dynamics of the mean prevalence of shedders in the default model and six sensitivity analyses. [file 1297-9716-44-28-S7.docx]
